# Predictive value of skin invasion in recurrent head and neck cancer patients treated by hypofractionated stereotactic re-irradiation using a cyberknife

**DOI:** 10.1186/s13014-015-0517-2

**Published:** 2015-10-15

**Authors:** Hideya Yamazaki, Mikio Ogita, Kengo Himei, Satoaki Nakamura, Gen Suzuki, Tadayuki Kotsuma, Ken Yoshida, Yasuo Yoshioka

**Affiliations:** Department of Radiology, Graduate School of Medical Science, Kyoto Prefectural University of Medicine, 465 Kajiicho Kawaramachi Hirokoji, Kamigyo-ku, Kyoto, 602-8566 Japan; CyberKnife Center, Soseikai General Hospital, 126 Kami-Misu, Shimotoba Fushimi-ku, Kyoto, Japan; Department of Radiotherapy, Fujimoto Hayasuzu Hospital, Hayasuzu 17-1, Miyakonojo, Miyazaki 885-0055 Japan; Department of Radiology, Japanese Red cross Okayama Hospital, Aoe 2-1-1, Kita-ku, Okayama, Okayama 700-8607 Japan; Department of Radiation Oncology, National Hospital Organization Osaka National Hospital, 2-1-14, Hoenzaka, Chuo-ku, Osaka, Osaka 540-0006 Japan; Department of Radiation Oncology, Osaka University Graduate School of Medicine, Suita, Osaka Japan

**Keywords:** Head Neck cancer, Reirradiation, Stereotactic radiotherapy, CyberKnife, Skin invasion

## Abstract

**Background:**

This study aimed to elucidate the influence of skin invasion in patients with recurrent head and neck cancer treated with re-irradiation using stereotactic radiotherapy.

**Materials:**

We reviewed 104 patients treated using CyberKnife in four institutions.

**Results:**

Nine cases of skin invasion were recognized (8.6 %). Larger tumors tended to exhibit skin invasion. The skin invasion (+) group showed a lower response rate (0/9, 0 %) than the skin invasion (−) group (56/95, 59 %) (*p* = 0.002). The skin invasion (+) group showed lower local control (LC) and progression free survival (PFS) rates, both 0 % at 6 months, than the skin invasion (−) group, which had a LC of 69 % (*p* = 0.0001) and a PFS of 48 % at 1 year (*p* = 0.0157). Median survival time and one-year survival rates for the skin invasion (+) and (−) groups were 6.6 vs. 15.3 months and 14 % vs. 59 % (*p* = 0.0005), respectively. No patient with skin invasion survived more than 14.4 months. The percentage of patients who developed grade 3 or higher toxicity was 44 % in the skin invasion (+) group and 18 % in the skin invasion (−) group (*p* = 0.14).

**Conclusions:**

Skin invasion is an important predictor of poor prognosis in recurrent head and neck cancer after re-irradiation with stereotactic radiation therapy.

## Background

Advanced radiotherapy techniques, i.e., stereotactic body radiation therapy (SBRT), intensity-modulated radiation therapy (IMRT), image-guided external radiotherapy, and new chemotherapeutic agents have improved the outcomes of unresectable head and neck cancer treatments [1, 2]. However, locoregional failure remains a major obstacle and requires further treatment. Unfortunately, one-third of the patients are eligible for salvage surgery [3]. Chemotherapy is frequently preferred, yet it results in less than 9 months of median survival [4]. With the advancements of modern radiation technique, re-irradiation has become a fascinating optional therapy using advanced technologies, i.e., IMRT and/or SBRT. The image-guided stereotactic radiotherapy system, CyberKnife, enables to deliver precise doses over short treatment periods [5–9]. Several institutions, including ours, reported the outcome and toxicity of re-irradiation using CyberKnife hypofractionated SBRT [5–9]. We have experienced lethal carotid blowout syndrome (CBOS) and found that skin invasion is an ominous predictor of CBOS-related death after CBOS [9]. These findings prompted us to investigate tumor factors, i.e., the presence of ulceration, in the prognosis of head and neck cancer patients [10]. However, skin invasion is rare; thus, it was difficult to assess its role simultaneously. We examined multi-institutional charts to identify the cases with skin invasion. The aim of this study was to examine the role of skin invasion in tumor control and toxicity after re-irradiation using CyberKnife SBRT for head and neck cancer patients.

## Materials and methods

**Fig. 1:**
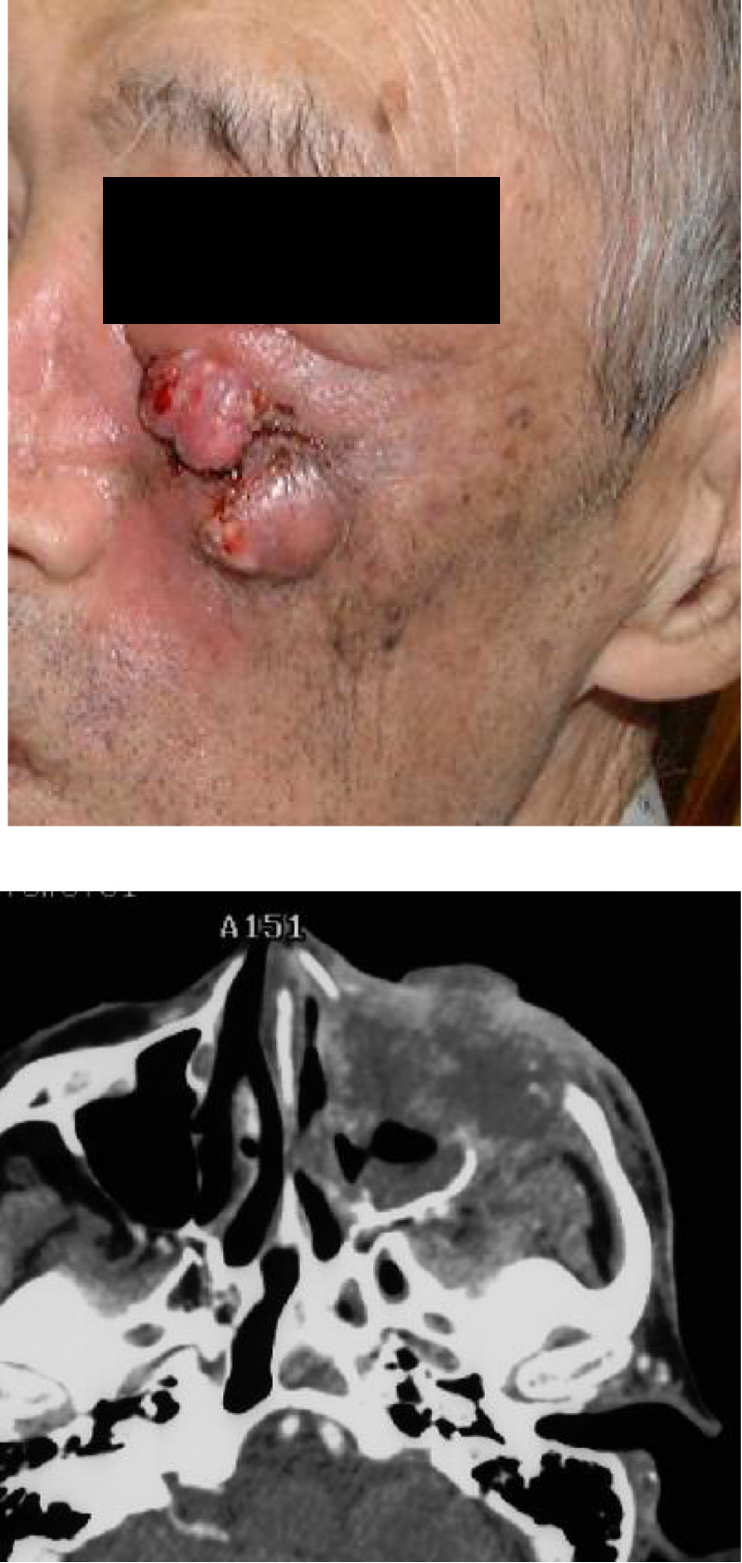
Presentation of a case associated with skin invasion. An 83 year old man with diagnosis of carcinoma maxillary sinus (T3N0) underwent radiotherapy with 66 Gy in 33 fractions associated with intra-arterial chemotherapy (cisplatin). A recurrent tumor was detected at the primary site eight months later. He then received 50 Gy in 25 fractions of re-irradiation; however, he developed another 2nd recurrence six months later. CyberKnife hypofractionated stereotactic radiation therapy was performed with 25.04 Gy in 8 fractions. The tumor exhibited skin invasion with larger PTV (124 cm^3^). He developed local recurrence with skin fistula 3 months after treatment and died 10 months later

We retrospectively reviewed medical records of patients who underwent CyberKnife SBRT (Accuray; Sunnyvale, CA, USA) at four hospitals (Soseikai General Hospital, Osaka University Hospital, Fujimoto Hayasuzu Hospital, and Okayama Kyokuto Hospital) during 2000–2010. Among 200 head and neck cancer patients who received reirradiation for residual or recurrent head and neck cancer, only those patients who satisfied the following criteria were included: image evaluation including computed tomography (CT) and/or Magnetic resornance imaging (MRI) before SBRT (Fig. 1) to confirm the presence or absence of skin invasion and had completed a course of radical treatment, including radiotherapy at ≥ 40 Gy (EQD2 prescribed in below), with or without chemotherapy and surgery. A total of 104 patients were eligible for assessment. The first course of radiotherapy was delivered by conventional technique using a linear accelerator. At the time of recurrence or residual disease, SBRT reirradiation was performed using the CyberKnife system. Patients received a median dose of 30 Gy (range, 15–39 Gy) over a median of 5 fractions (range, 3–8 fractions) that were prescribed at D90, D95, or a marginal dose. D90 (D95) was defined as a minimum dose covering 90 % (95 %) of the planning target volume (PTV). The marginal dose prescription was defined by percent (100 %, maximum dose) of the isodose curve covering the PTV. Median cumulative dose (dose from the first course of radiation and dose received from the second course of radiation) was 91Gy (range: 62-146Gy). Interval between the first course of radiation and the second course of radiation was 14.5 months in median value (range: 0.7-1180 months). The presence of skin invasion was identified by imaging analysis CT and/or MRI such as computed tomography and/or magnetic resonance imaging (contrast-enhanced if required). A biologically equivalent dose (BED) was calculated into equivalent 2-Gy fractions (EQD2) using the linear quadratic model: EQD2 = prescription dose × (α/β + dose per fraction)/(α/β + 2), where α/β = 10 for tumors and 3 for organs at risk. All studies on humans described in the present manuscript were carried out in accordance with the Helsinki Declaration. Written informed consent was obtained from the patients for publication of this data and the accompanying images.

### Statistical analysis

All statistical analyses were performed using Stat-view 5.0 statistical software (SAS Institute, Inc., Cary, NC, USA). The percentage values were analyzed using the *χ*^2^ test, and values were compared using Mann–Whitney U analysis. Cumulative incidences were estimated by the Kaplan–Meier method. The durations were calculated from the first day of CyberKnife SBRT. Cut-off value was set at average or median value of each variable if otherwise stated. All analysis used a *p*-value < 0.05 level of significance.

## Results

**Fig. 2:**
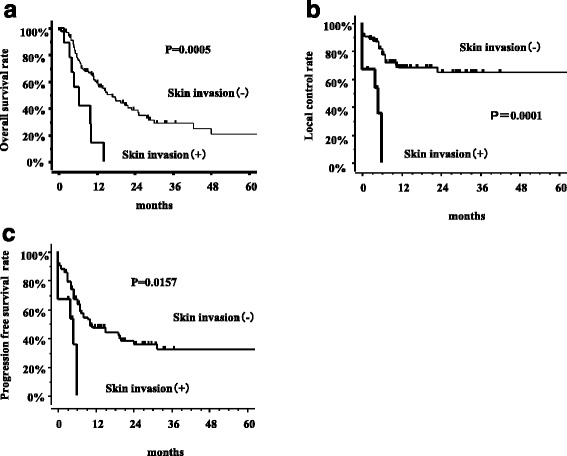
Influence of skin invasion. The thick line represents the skin invasion (−) group and the thin line represents the skin invasion (+) group. **a** Local control rates according to the presence of skin invasion. **b** Progression-free survival rates according to the presence of skin invasion. **c** Overall survival rates according to the presence of skin invasion

The median follow-up time for the surviving patients after SBRT was 13.7 months (range 1–122 months). There were significant differences in PTV between the skin invasion (+) and (−) cases (Table 1). In other words, larger tumors tended to exhibit more skin invasion. The skin invasion (+) group showed a lower response rate (0/9, 0 %) than the skin invasion (−) group (56/95, 59 %) (*p* = 0.002) (Table 2). (Table 2) Notably, no patient in the skin invasion (+) group obtained complete or partial remission. The skin invasion (+) group had both a local control (LC) and a progression-free survival (PFS) rate of 0 % at 6 months, whereas the skin invasion (−) group had a LC rate of 69 % (*p* = 0.0001) and a PFS rate of 47 % at 1 year (*p* = 0.0157) (Fig. 2). Median survival time and one-year survival rates for the skin invasion (+) and (−) groups were 6 vs. 15.3 months and 16.7 % vs. 57 % (*p* = 0.0005), respectively (Fig. 2). No patients with skin invasion survived more than 14.4 months. Therefore, there were statistical significant differences in all the prognostic indicators (initial response rate, LC, PFS and overall survival) between the skin invasion (+) and (−) cases (Fig. 2), indicating poor prognosis for patients with skin invasion. The local control rate, progression-free survival rate, and overall survival rate at 1 year were 58, 31, and 47 %, respectively in the treatment interval ≦30 months group (*n* = 68) and 76 % (*p* = 0.11), 70 % (*p* = 0.002), and 66 % (*p* = 0.02) in the treatment interval 30 months < group (*n* = 36).

**Table 1:** Comparison of patients characteristics, treatment factors and outcome between skin invasion (+) and (−) patients

Variables	Strata	Skin invasion (−)		Skin invasion (+)		*p*-value
		*n* = 95		*n* = 9		
		No. or mean +/− SD, median (range)	(%)	No. or median (range)		
Age		64 (43–88)		61 (56–83)		0.65
Gender	Female	26	(27 %)	1	(11 %)	0.5
	Male	69	(73 %)	8	(89 %)	
Disease	Nasopharyngeal cancer	41	(43 %)	0	(0 %)	0.12
	Oropharyngeal cancer	19	(20 %)	2	(22 %)	
	Hypopharyngeal cancer	9	(9 %)	2	(22 %)	
	Oral cancer	10	(11 %)	2	(22 %)	
	Nasal/pranasal	16	(17 %)	3	(33 %)	
Irradiated area	Primary	73	(77 %)	5	(56 %)	0.31
	Lymph node	22	(23 %)	4	(44 %)	
rT stage	T0	12	(13 %)	2	(22 %)	0.25
	T1	18	(19 %)	0	(0 %)	
	T2	9	(9 %)	1	(11 %)	
	T3	11	(12 %)	3	(33 %)	
	T4	30	(32 %)	3	(33 %)	
	NA	15	(16 %)	0	(0 %)	
rN stage	N0	69	(73 %)	4	(44 %)	0.32
	N1	18	(19 %)	3	(33 %)	
	N2	5	(5 %)	1	(11 %)	
	N3	1	(1 %)	0	(0 %)	
	NA	2	(2 %)	1	(11 %)	
Ulceration	(−)	74	(78 %)	5	(56 %)	0.27
	(+)	21	(22 %)	4	(44 %)	
Surgical history	(−)	55	(58 %)	3	(33 %)	0.28
	(+)	40	(42 %)	6	(67 %)	
Planning target volume	cm3	26.2 (0.9–339)		64.6 (5.2–241)		**0.04**
Interval	months	16.0 (1–1180)		8.3 (5.1–44)		0.1
Dose	EQD2 (a/b = 10)	41.9 (18.9–74.7)		34.6 (31.2–57.8)		0.12
Cumulative dose	EQD2 (a/b = 10)	117.1 (62–192)		105. 4 (96.6–130)		0.09
Initial tumor response	CR + PR	55	(59 %)	0	(0 %)	**0**
	SD + PD	38	(41 %)	9	(100 %)	
Toxicity	Grade 0–2	78	(82 %)	5	(56 %)	0.14
	Grade 3-	17	(18 %)	4	(44 %)	

*CR* complete reponse, *PR* partial response, *NC* no change, *PD* progressive disease

Bold values indicate statistically significance

**Table 2:** CBOS according to carotid invasion and skin invasion

Variables			Pt. NO.	CBOS ratio
Carotid invasion	Skin invasion	CBOS		
≤180 degree	-	-	35	0 %
		+	0	
	+	-	5	0 %
		+	0	
180 degree <	-	-	39	19 %
		+	9	
	+	-	2	50 %
		+	2	
Not available	-	-	12	0 %
		+	0	
	+	-	0	0 %
		+	0	

*CBOS* carotid blow-out syndrome

### Toxicity

A total of 21 patients experienced grade 3 or higher toxicities (20 %). Among them, CBOS occurred in 11 patients and resulted in nine deaths, whereas the other two patients recovered following intervention. All lethal toxicities were due to CBOS. CBOS developed in 22 % of the patients in the skin invasion (+) group (2/9), and in only 9 % of those in the skin invasion (−) group (9/95) (*p* = 0.24). Two cases of CBOS with skin invasion could not be salvaged and showed poor prognosis (1.6 months and 13.6 months after reirradiation) resulted in death. There are no CBOS case in patients with carotid invasion ≦180° (Table 2). In patients with carotid invasion 180°<, 50 % of patients showed CBOS with skin invasion, whereas 19 % without (Table 2). We could not find statistical significance in prescribed dose between CBOS (median 87 Gy: range 75–109 Gy) and non-CBOS group (median 92 Gy: range 66–146 Gy). Other grade 3 or higher toxicities included two cases of mucositis requiring percutaneous endoscopic gastrostomy, two cases of lateral lobe necrosis (one grade 4), five fistulas, one bone necrosis, one soft tissue necrosis, one visual disturbance, and one ulceration. The percentage of patients who developed grade 3 or higher toxicity was 44 % in the skin invasion (+) group and 18 % in the skin invasion (−) group (*p* = 0.14). Although this difference was not statistically significant, the skin invasion (+) group showed three times more frequent severe toxicity of grade 3 or higher, which implied a vulnerability to suffer severe toxicities in the skin invasion (+) group.

## Discussion

This is the first study to examine the role of skin invasion in recurrent head and neck cancer patients who underwent re-irradiation with CyberKnife SBRT. Skin invasion is an established risk factor for newly diagnosed head and neck cancer patients. It is regarded as a T4 category in the TNM classification of various types of head and neck cancers, i.e., oral and nasal/paranasal cancers [11]. Therefore, it is plausible that skin invasion may be nasalidentified as a significant predisposing factor after re-irradiation also. In a previous study, we observed poor prognosis in post-operative patients with skin invasion in case of CBOS after SBRT [7] (Fig. 1). This observation prompted us to explore the role of skin invasion in re-irradiation. As highfound that skin invasion is a predictor of poor prognosis after re-irradiation, and no patients with skin invasion survived more than 14.4 months after SBRT. None of the patients with skin invasion obtained a partial response or a better clinical outcome after SBRT, and all cases recurred within 6 months. The poor tumor response and local control could be important findings to explain the poor survival rate in these patients.

We did not find a statistically significant correlation between skin invasion and CBOS nor toxicities. It can be predicted that skin invasion itself does not always imply tumor invasion into the carotid artery. For example, it is rare to find carotid invasion in nasal or paranasal cancer patients with skin invasion (Fig. 1). On the other hand, as there is a close relationship between skin invasion and tumor volume, larger tumors tend to show skin invasion and, at the same time, may pose higher risk of serious toxicities. In general, the risk of higher toxicity should be particularly considered when evaluating the indications for re-irradiation. Our data of 44 % of patients with grade ≥ 3 toxicity in the skin invasion (+) group could be high enough to prompt to consider more strict eligibility criteria for re-irradiation in patients with skin invasion. Several attempts have been made to reduce toxicity. Instead of daily SBRT, treatment on alternate days could be an option [5-12]. Conventional dose fractionation (1.8 − 2 Gy/fraction) also could reduce toxicity. IMRT is now being explored [13]; however, there is little data in cases with skin invasion. In addition, even IMRT caused a cumulative incidence of late grade ≥ 3 toxicity of 23, 27, and 66 % at 1, 2, and 5 years, respectively. In 4 patients, death was attributed toxicity including fatal bleeding (*n* = 2), aspiration pneumonia (*n* = 1), and skin necrosis (*n* = 1). Therefore, risk factor assessment including tumor factors, i.e., ulceration and skin invasion, should be included in evaluating patient eligibility for re-irradiation.

This study had several limitations. First, because of the small number of patients with skin invasion, it is difficult to state clearly the role of this factor without confounding variables. In our analysis, skin invasion was related to tumor size, which indicates tumor aggressiveness. In addition, this study was a retrospective analysis that included a small number of patients with a short follow-up period, thus selection- and physician-based biases may exist. Therefore, these results should be confirmed in a prospective trial with a larger number of patients and longer follow-up period.

In conclusion, skin invasion is an important predictive factor for the prognosis of recurrent head and neck cancer after SBRT.
